# Engaging civil society with health research

**DOI:** 10.1098/rsnr.2016.0037

**Published:** 2016-09-14

**Authors:** Mary Madden

**Affiliations:** School of Healthcare, Faculty of Medicine and Health, Baines Wing, University of Leeds, Leeds LS2 9JT, UK

The move to an open access model in contemporary health research raises questions about the role of the scientific journal and its engagement with civil society in shaping the research agenda, as well as highlighting tensions between the public interest in science and doing science that is in the public interest. If openness is seen as fundamental to the advancement of scientific discovery, a pay-to-view model of publishing militates against this. Publicly funding science implies a responsibility to share the results and benefits with the public who fund it. Health research, and its availability, is the foundation of the right to health in the World Health Organization Constitution. Erecting financial barriers can mean that health research is not available to those who need it most.

The momentum towards free and open access publishing is making research literature available to the public but there are limitations, restrictions and exclusions. The assumption that all research will be directly funded at a high enough level to pay fees for article processing charges comes in at a time when science and healthcare budgets are under particular pressure. Even with open access, health research is not free, but a changing commodity in markets of publishing, journalism, the university sector, health services, pharmaceutical and medical device industries. Universities make economic and strategic decisions about which academic papers they should fund in an academic market focused on the competition of research assessment/excellence frameworks. Academics are assessed through what and where they publish. The imperative to publish is not necessarily the same as doing good science or pursuing the public interest. The introduction and widespread adoption of systematic review and meta-analytic methods to summarize research provides growing evidence that publication in peer-reviewed journals does not guarantee a study's validity.^1^ The Cochrane Collaboration regularly excludes from evidence 50–75% of published studies because of poor design or reporting which undermines the trustworthiness of their conclusions.^2^ An appetite for new breakthroughs, despite the importance of null results for health care knowledge, leads to publication bias in favour of positive results, further fuelling expectations.

Research is only really accessible if it is intelligible to the audiences who may benefit from it. But who actually reads journals? Where do people get their health information? Who are the contemporary popularizers of science and are these trustworthy and unbiased sources? While patients, their families and the public are a potential audience, information inequality maps on to growing health inequality globally and locally. Once a journal is opened, what of its readability and style? The move to produce ‘plain language’ summaries arguably exchanges highly technical language for differently dull and unengaging prose.

Citizen science implies active engagement, yet the public and health professionals are often addressed as passive consumers of evidence from researchers. The James Lind Alliance identifies a mismatch between the priorities of academics and clinicians and those of people with direct experience of a health condition.^3^ The health research narrative tends to be driven by medicine rather than nursing, midwifery, physiotherapy and other allied health professions. A pervasive ‘deficit model’ implies that all public and professional scepticism of science is unfounded and that there is a need for corrective communication by experts rather than a need to encourage broader debate that attends to those concerns. At the same time there is a growing recognition of patients or service users as the site and source of evidence.

National Health Service research funding is now contingent on demonstrating patient and public involvement (PPI) and impact, defined as the contribution research makes to society via the steps researchers have taken to increase the chances of potential beneficiaries benefiting from their work. Involvement and engagement strategies can potentially dramatically transform how researchers engage with the public, or alternatively serve as technologies of legitimacy (reinforcing the status quo), re-positioning non-academic participants as raw materials for auditable research rather than equal partners in processes of co-production. PPI in research is defined as research carried out ‘with’ or ‘by’ members of the public, rather than being directed ‘to’, or being ‘about’ or ‘for’ them. Individual and group-based challenges to research originating from service users have in some cases resulted in fruitful research collaborations, e.g. in HIV and breast cancer. However, PPI remains conceptually and theoretically vague and in practice the distinction between involvement, engagement and participation is blurred. It has become an NHS research imperative on the basis that it improves methodological quality and relevance, although the actual empirical evidence base remains poor and reporting is predominantly descriptive rather than evaluative.^4^ The imperative is linked to the hope that actively involving patients and the public can help researchers improve recruitment, retention and relevance of the randomized controlled trials (RCTs) which are at the heart of research into treatment effectiveness. Those recruited to RCTs may not directly benefit and may experience burden or even harm.

Because research into PPI itself is not given high priority, it is not clear what effective PPI or co-production research processes look like, nor how to develop and maintain such a process over time. It has also been hard to find a place to publish PPI methodology. *Research Involvement and Engagement* is a new interdisciplinary, health and social care gold open access journal focusing on patient and wider involvement and engagement in research which is co-produced by key stakeholders, including patients, academics, policy makers and service users. *Research for All* is a new open access journal focusing on research that involves universities and communities, services or industries working together. Such journals are platforms for articles that may contribute to the evidence base for involvement and engagement. It remains to be seen whether they will replicate or depart from the ongoing problems for open access journals, not least the challenge of securing the funds to publish in them. Some veterans of the campaign to integrate citizen involvement in health research view the idea of separate PPI journals as a potentially retrograde step.

Citizen science can be spontaneous, invited or sponsored, with citizens engaging as compliant data gatherers, sources of data or legitimization, or as user-leaders who are makers and challengers of theory.^5^ The changing role of the scientific journal is an important part of the social and political context of health care knowledge production and broader cultural conversations and contestations about health equity, democracy and the rights and responsibilities of citizenship.

## Notes

1 J. P. A. Ioannidis, ‘Why most published research findings are false’, *PLoS Med* **2**, e124 (2005).

2 S. B. Soumerai, D. Starr and S. R. Majumdar, ‘How do you know which health care effectiveness research you can trust? A guide to study design for the perplexed’, *Prev. Chronic Dis.* **12**, 150–187 (2015).

3 S. Crowe, M. Fenton, M. Hall *et al.*, ‘Patients’, clinicians' and the research communities' priorities for treatment research: there is an important mismatch', *Research Involvement and Engagement* **1**, 2 (2015), https://researchinvolvement.biomedcentral.com/articles/10.1186/s40900-015-0003-x (accessed on 15 July 2016).

4 K. Staley, *Exploring impact: public involvement in NHS, public health and social care research* (INVOLVE, Eastleigh, 2009).

5 P. Wehling, W. Viehöver and S. Koenen (eds), *The public shaping of medical research: patient associations, health movements and biomedicine* (Routledge, London, 2015).

